# The diagnostic and prognostic utility of blood metagenomic next-generation sequencing for invasive pulmonary aspergillosis

**DOI:** 10.1128/spectrum.03384-25

**Published:** 2026-04-17

**Authors:** Yuhui Chen, Xinzhu Tang, Sifen Lu, Liyan Guo, Laduona Wang, Lang Min, Ting Niu, Yongzhao Zhou

**Affiliations:** 1 Department of Hematology, Institute of Hematology, West China Hospital, Sichuan University 12530, Chengdu, China; 2 West China School of Medicine, Sichuan University, Chengdu, China; 3 Department of Hematology, West China Lecheng Hospital of Sichuan University 12530, Qionghai, China; 4 Precision Medicine Center and Breathing and Comorbidities Precision Medicine Key Laboratory of Sichuan Province, West China Hospital, Sichuan University 12530, Chengdu, China; 5 State Key Laboratory of Biotherapy, Collaborative Innovation Center of Biotherapy, West China Hospital, Sichuan University 12530, Chengdu, China; 6 National Facility for Translational Medicine (Sichuan), West China Hospital, Sichuan University 12530, Chengdu, China; 7 Integrated Care Management Center, West China Hospital, Sichuan University 12530, Chengdu, China; 8 Department of Pulmonary and Critical Care Medicine, West China Hospital, Sichuan University 12530, Chengdu, China; Central Texas Veterans Health Care System, Temple, Texas, USA

**Keywords:** Metagenomic next-generation sequencing (mNGS), invasive pulmonary aspergillosis, biomarker, prognosis

## Abstract

Differentiating invasive pulmonary aspergillosis (IPA) from colonization in patients with *Aspergillus*-positive blood metagenomic next-generation sequencing (mNGS) remains a clinical challenge. This study aims to evaluate the diagnostic and prognostic value of blood mNGS-derived fungal load (reads per million [RPM]) and two key serological biomarkers (galactomannan [GM] and 1,3-β-D-glucan [BDG]) in distinguishing these two entities. This retrospective study enrolled 95 patients with *Aspergillus* detected by blood mNGS, stratified into infection (*n* = 60) and colonization (*n* = 35) groups using modified EORTC/MSGERC criteria. We analyzed clinical characteristics, co-infection spectra, and serological biomarkers (GM and BDG). Diagnostic performance was evaluated via receiver operating characteristic (ROC) analysis, and prognostic factors for 28-day mortality were identified using least absolute shrinkage and selection operator-Cox regression. Distinct co-infection patterns were observed between groups: the infection group was dominated by polymicrobial co-infections, including clinically significant pathogens such as *Acinetobacter baumannii*, *Klebsiella pneumoniae*, *Mucor* spp., and *Human cytomegalovirus*; in contrast, the colonization group primarily featured single viral co-infections. While mNGS effectively detected *Aspergillus*, RPM alone showed limited ability to discriminate infection from colonization, with area under the curves (AUCs) ranging from 0.406 to 0.657 across patient groups. The optimal RPM cutoff varied substantially by immune status, being highest in immunocompetent patients (RPM cutoff: 1.77). Diagnostic performance significantly improved when RPM was integrated with GM (AUC up to 0.900 at a cutoff of 0.36 optical density index) or BDG (AUC up to 0.881 pg/mL), particularly in immunocompetent individuals. RPM also correlated with albumin, hemoglobin, platelet counts, and lymphocyte counts (all *P* < 0.05). Multivariate analysis identified reversed halo sign (hazard ratio [HR] = 2.143), decreased ratio of partial pressure of arterial oxygen to fraction of inspired oxygen (PaO_2_/FiO_2_; HR = 1.361), and elevated lactate dehydrogenase (HR = 1.055) as independent predictors of 28-day mortality. Blood mNGS demonstrates high sensitivity for detecting *Aspergillus* but requires integration with serological biomarkers to differentiate IPA from colonization. The RPM can offer prognostic utility. A multimodal strategy is crucial for early diagnosis and improving outcomes in high-risk patients.

**IMPORTANCE:**

First large-scale validation of blood mNGS for invasive pulmonary aspergillosis diagnosis—this study represents the first sizable cohort systematically evaluating blood metagenomic next-generation sequencing (mNGS) for distinguishing invasive pulmonary aspergillosis from colonization, addressing a critical gap in non-invasive diagnostic approaches for critically ill patients. Comprehensive *Aspergillus* co-infection profiling—we identified distinct co-infection patterns, with the infection group showing significantly higher rates of polymicrobial infections, providing crucial insights into co-infection dynamics in *Aspergillosis*. Optimized diagnostic integration strategy—our findings demonstrate that while mNGS-derived reads per million alone show limited diagnostic value, their integration with serological biomarkers significantly improves performance, establishing a clinically relevant multimodal diagnostic framework. Robust prognostic stratification model—through least absolute shrinkage and selection operator-Cox regression, we established a validated prognostic model identifying reversed halo sign, decreased PaO_2_/FiO_2_, and elevated lactate dehydrogenase as independent predictors of 28-day mortality, providing clinically actionable tools for risk stratification.

## INTRODUCTION

*Aspergillus*, a ubiquitous fungus, frequently colonizes the respiratory tract of immunocompromised or critically ill patients (1). Invasive pulmonary aspergillosis (IPA) and *Aspergillus* colonization represent two distinct clinical entities with profoundly different implications for patient management and prognosis. Discriminating between them remains a critical challenge in clinical practice (2, 3). In immunocompromised hosts, colonization can progress to invasive infection, which is associated with severe morbidity and mortality rates exceeding 50% in high-risk populations (4). Thus, accurate and timely differentiation is essential to avoid unnecessary antifungal therapy or delayed treatment (5).

Conventional diagnostic methods for IPA have several limitations. Although culture and microscopic examination of lower respiratory tract specimens or lung biopsy tissue are considered gold standards for identification, they suffer from low sensitivity (often <50%) and long turnaround times, which can delay treatment decisions (6, 7). Moreover, invasive procedures such as bronchoscopy are often not well tolerated by severely ill patients. The galactomannan (GM) assay is an important tool for IPA diagnosis, but its diagnostic value varies with specimen type. A meta-analysis supporting current guidelines indicates that GM testing of bronchoalveolar lavage fluid (BALF) has superior sensitivity compared to blood testing for diagnosing invasive aspergillosis (8). Other serological biomarkers, such as 1,3-β-D-glucan (BDG), lack species specificity and are unable to reliably distinguish *Aspergillus* from other fungal pathogens (9). *Aspergillus*-specific IgG serology shows some diagnostic value for chronic infections but is less useful in acute or invasive settings. Polymerase chain reaction (PCR) offers rapid results but is hampered by a lack of standardization, undefined thresholds, and limited coverage of *Aspergillus* species, especially in blood samples (10). Imaging features such as the halo sign or air crescent sign are typical of IPA but are often absent in critically ill patients, reducing their diagnostic utility in intensive care settings (11).

Metagenomic next-generation sequencing (mNGS) has emerged as a transformative tool in infectious disease diagnostics, offering unbiased detection of pathogens without prior knowledge of the causative agent (12). Its application in respiratory infections, including IPA, has been validated in multiple cohorts, with studies demonstrating superior sensitivity compared to conventional methods, especially in identifying mixed infections or rare species (13). For *Aspergillus*, mNGS of BALF has shown promise in differentiating infection from colonization, with an area under the curve (AUC) of 0.894 and an optimal threshold of 23 reads per 10 million (RPTM) in a recent study (3). However, most research has focused on BALF specimens, and the diagnostic performance of blood-based mNGS for *Aspergillus*, particularly in distinguishing infection from colonization, remains underexplored, despite its potential as a non-invasive alternative for critically ill patients unable to undergo bronchoscopy (14, 15).

Against this backdrop, our study aims to address these critical knowledge gaps. Specifically, we sought to characterize the spectrum of co-infections in both groups and their clinical implications and to identify risk factors associated with 28-day mortality via least absolute shrinkage and selection operator (LASSO)-Cox regression. By addressing these objectives, we aim to refine the clinical utility of blood mNGS in *Aspergillus* diagnostics and improve prognostic stratification, ultimately guiding more precise and timely patient management.

## MATERIALS AND METHODS

### Study design and participants

**Fig 1:**
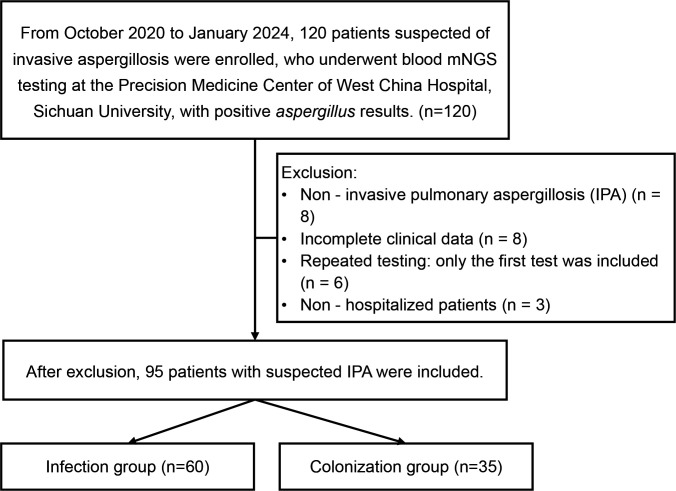
Patient enrollment and study flow diagram.

This retrospective study initially screened 120 patients with *Aspergillus* detected by blood mNGS at West China Hospital, Sichuan University, between October 2020 and January 2024. After excluding patients with incomplete clinical data or inconsistent diagnostic classification, 95 eligible patients were finally enrolled (Fig. 1). Two experienced clinicians classified patients into two groups using a modified framework based on the 2019 EORTC/MSGERC Consensus Definitions (16): the *Aspergillus* infection group (*n* = 60) included patients meeting criteria for proven, probable, or possible IPA (proven IPA required histopathological evidence or a positive culture from a sterile site; probable IPA required the presence of host factors, clinical features, and mycological evidence; and possible IPA required host and clinical criteria in the absence of mycological confirmation), while the *Aspergillus* colonization group (*n* = 35) consisted of patients with positive blood mNGS for *Aspergillus* but no evidence of infection (absence of host factors, characteristic clinical manifestations, or supportive mycological findings). Detailed diagnostic assessments for each patient are provided in Table S1.

### Data collection

Clinical data were extracted from electronic medical records, encompassing demographic characteristics, clinical symptoms and signs, laboratory findings (including GM test [serum/BALF], BDG, and blood mNGS-derived reads per million [RPM] for *Aspergillus*), imaging findings (chest computed tomography features), treatment regimens, and outcomes.

### Antigen detection assay

GM assay: Serum and BALF samples were tested using the Platelia *Aspergillus* Ag kit (Bio-Rad, France) in strict accordance with the manufacturer’s instructions. Briefly, microtiter plates coated with anti-GM monoclonal antibody (EB-A2) were incubated with diluted samples (1:10 for serum, undiluted for BALF) at 37°C for 1 hour. After washing, peroxidase-conjugated EB-A2 antibody was added and incubated for 1 hour, followed by substrate solution (tetramethylbenzidine) incubation for 30 minutes. The reaction was stopped with 1 M sulfuric acid, and absorbance was measured at 450 nm using a microplate reader. The optical density index (ODI) was calculated as the ratio of sample absorbance to the cutoff calibrator absorbance, with positive thresholds set at ODI ≥0.5 (serum) and ODI ≥1.0 (BALF) (15).

BDG assay: Serum samples were analyzed using the Fungitell assay (Associates of Cape Cod Inc., USA) following the manufacturer’s protocol. Briefly, 50 μL of preheated (70°C for 10 minutes) serum was mixed with 100 μL of Fungitell reagent (containing G factor from Limulus amebocyte lysate) and incubated at 37°C for 1 hour. The absorbance at 405 nm was measured, and BDG concentrations (pg/mL) were calculated using a standard curve generated with calibrators (0, 20, 80, 160, and 320 pg/mL). A positive result was defined as BDG ≥80 pg/mL (8).

### Definition

Immunocompromised status was defined as the presence of any of the following conditions: active hematological malignancy or solid tumor receiving chemotherapy; acquired immunodeficiency syndrome with CD4^+^ count <200 cells/μL; profound CD4^+^ lymphopenia from other causes; or receipt of significant pharmacologic immunosuppression. This included treatment with calcineurin inhibitors, TNF-α inhibitors, or corticosteroids at a dose equivalent to prednisone ≥20 mg per day for ≥4 weeks.

### Blood mNGS procedures

Blood samples were collected from patients and submitted to the Precision Medicine Key Laboratory of Sichuan Province and Precision Medicine Center for mNGS testing. Genomic DNA was extracted using the TIANamp Micro DNA Kit (cat. no. DP316; TIANGEN BIOTECH, Beijing, China) following the manufacturer’s instructions. Library preparation was performed using the MGIEasy DNA Library Prep Kit (MGI Tech Co., Ltd., Shenzhen, China) according to the kit protocol. Library quality control was performed using an Agilent 2100 Bioanalyzer to verify fragment sizes (target range: 200–300 bp), and the Qubit dsDNA HS Assay Kit (Thermo Fisher Scientific, Inc., Waltham, Massachusetts, USA) was used to quantify library concentrations. High-throughput sequencing was conducted on the MGI2000 (MGI Tech Co., Ltd., Shenzhen, China) platform to generate mNGS data. Raw sequencing data were processed to filter out low-quality reads and adapter contaminants. The cleaned data were then aligned against the human reference genome using BWA software (https://bio-bwa.sourceforge.net/) to exclude host sequences (17). After further removal of low-complexity sequences, the remaining reads were mapped to the BGI Microbial Reference Database (PMDB), which includes 6,350 bacterial, 1,064 fungal, 4,945 viral, and 234 parasitic species (17). Contamination and implausible results were excluded by combining data from sterile normal saline negative controls. These controls were sterile 0.9% sodium chloride solution free of microbial nucleic acid contamination, purchased from Chengdu Kelun Pharmaceutical Co., Ltd., Chengdu, China, and processed in parallel with patient blood samples through all steps (sample collection, nucleic acid extraction, library construction, and sequencing) to monitor potential cross-contamination. The exclusion was conducted with the following criteria: (i) species with RPM values less than threefold that in negative control samples; (ii) commonly recognized contaminant species; (iii) species with fewer than three reads at the species level; (iv) species with uneven read distribution across the reference genome (excessively concentrated reads may represent random fragments rather than true sequences); (v) common commensal bacteria of the respiratory tract as reported in the literature; (vi) species showing strong positive signals in other samples of the same batch (with >10,000 reads or significantly more reads than other samples), which were excluded after considering sample extraction order; and (vii) non-pathogenic environmental species. Following contamination exclusion, bacterial species were further filtered by ranking genus-level read counts, retaining only the top 10 genera and the top 2 species within each genus (18). However, highly pathogenic species (e.g., *Klebsiella pneumoniae*) were retained regardless of this ranking as long as they met the above exclusion criteria. Fungi and viruses were retained if they passed the exclusion criteria. For parasites, due to their larger genome size and higher similarity to the human genome, an additional criterion (read count > 10) was applied alongside the aforementioned exclusion criteria (18).

### Statistical analysis

All analyses were conducted in R (version 4.4.1) with key packages including tableone for baseline table generation, pROC for ROC curve analysis, glmnet for LASSO regression, and survival for Cox proportional hazards modeling. Associations between blood mNGS-derived *Aspergillus* load (RPM) and clinical parameters were assessed using Spearman’s rank correlation analysis, with correction for multiple comparisons where appropriate. GraphPad Prism (version 8) was used for visualization of infection distribution patterns. Continuous variables were assessed for normality using Shapiro-Wilk tests, with normally distributed data presented as mean ± standard deviation and non-normal data as median (interquartile range). Categorical variables were reported as counts (percentages). For ROC curve analysis, the pROC package was employed to calculate the AUC with 95% confidence intervals (CIs), and optimal cut-off values were determined by maximizing the Youden index. LASSO regression was implemented using the glmnet package with 10-fold cross-validation to screen for high-risk factors, where the optimal lambda (λ) was selected based on minimum cross-validation error. Cox proportional hazards regression was performed using the survival package to evaluate the association between selected factors and 28-day mortality, with hazard ratios (HRs) and 95% CIs reported. The proportional hazards assumption was verified using Schoenfeld residuals. Statistical significance was defined as two-tailed *P* < 0.05 unless otherwise specified.

## RESULTS

### Demographic and clinical characteristics

A total of 95 patients with *Aspergillus* species detected by blood mNGS were enrolled in this study and categorized into an infection group (*n* = 60) and a colonization group (*n* = 35; Fig. 1). Significant differences were observed between the groups across multiple clinical domains (Table 1). Patients in the infection group exhibited a higher prevalence of hypoxemia (95.0% vs. 65.7%, *P* < 0.001). Radiologically, pulmonary involvement (100.0% vs. 82.9%, *P* = 0.004), bilateral involvement (100.0% vs. 82.9%, *P* = 0.004), ground-glass opacity (73.3% vs. 42.9%, *P* = 0.006), patchy consolidation (65.0% vs. 31.4%, *P* = 0.003), and pleural effusion (56.7% vs. 28.6%, *P* = 0.015) were all more common in the infection group. Respiratory support requirements also differed significantly (*P* = 0.007), with the infection group having higher rates of invasive mechanical ventilation (50.0% vs. 25.7%) and fewer patients requiring no oxygen supplementation (8.3% vs. 34.3%). Prognostic and severity indicators consistently favored the colonization group: the infection group had significantly higher 28-day mortality (65.0% vs. 28.6%, *P* = 0.001), lower median PaO_2_/FiO_2_ (167.10 vs. 450.00 mmHg, *P* < 0.001), higher intensive care unit (ICU) admission rates (66.7% vs. 31.4%, *P* = 0.002), longer median ICU stays (5.00 vs. 0.00 days, *P* = 0.001), longer median hospital stays (13.00 vs. 5.00 days, *P* = 0.048), and higher median sequential organ failure assessment (SOFA) scores (11.00 vs. 4.00, *P* < 0.001). Numerous laboratory indices, including neutrophil percentage, lactate dehydrogenase (LDH), ALB, GLB, AST, Scr, eGFR, BNP, PCT, and IL-6, showed significant differences (all *P* < 0.05).

**TABLE 1:** Baseline demographic and clinical characteristics of infection and colonization group^*a*^

	Overall, *N* = 95	Infection, *N*= 60	Colonization, *N* = 35	*P*-value
Sex = male (%)	62 (65.3)	43 (71.7)	19 (54.3)	0.135
Age (years), (median [IQR])	55.92 (18.32)	58.25 (15.52)	51.91 (21.99)	0.104
Pathogen species simultaneously detected by mNGS				
Bacteria (%)	47 (49.5)	33 (55.0)	14 (40.0)	0.231
Fungi (%)	21 (22.1)	17 (28.3)	4 (11.4)	0.097
Virus (%)	70 (73.7)	45 (75.0)	25 (71.4)	0.889
Others (%)	2 (2.1)	2 (3.3)	0 (0.0)	0.726
Underlying diseases (%)				0.173
Chronic respiratory diseases	10 (10.5)	9 (15.0)	1 (2.9)	
Hematologic diseases or solid organ tumor	25 (26.3)	11 (18.3)	14 (40.0)	
Immune-mediated inflammatory disease	9 (9.5)	6 (10.0)	3 (8.6)	
Organ failure	13 (13.7)	8 (13.3)	5 (14.3)	
Organ transplantation	8 (8.4)	5 (8.3)	3 (8.6)	
Others	30 (31.6)	21 (35.0)	9 (25.7)	
Immunosuppressant exposure (%)	18 (18.9)	11 (18.3)	7 (20.0)	1
Corticosteroids exposure (%)	25 (26.3)	16 (26.7)	9 (25.7)	1
Chemotherapy or radiation therapy (%)	20 (21.1)	10 (16.7)	10 (28.6)	0.266
HSCT or organ transplantation (%)	16 (16.8)	8 (13.3)	8 (22.9)	0.362
Diabetes mellitus (%)	27 (28.4)	21 (35.0)	6 (17.1)	0.104
Hypertension (%)	27 (28.4)	19 (31.7)	8 (22.9)	0.495
Chronic kidney disease (%)	20 (21.1)	16 (26.7)	4 (11.4)	0.135
Chronic lung diseases (%)	12 (12.6)	11 (18.3)	1 (2.9)	0.061
Chronic heart insufficiency (%)	25 (26.3)	19 (31.7)	6 (17.1)	0.19
Drinking (%)	19 (20.0)	13 (21.7)	6 (17.1)	0.79
Smoke (%)	28 (29.5)	22 (36.7)	6 (17.1)	0.075
Symptoms				
Fever (%)	73 (76.8)	45 (75.0)	28 (80.0)	0.76
Cough and sputum (%)	45 (47.4)	32 (53.3)	13 (37.1)	0.19
Hemoptysis (%)	7 (7.4)	4 (6.7)	3 (8.6)	1
Hypoxemia (%)	80 (84.2)	57 (95.0)	23 (65.7)	<0.001
Chest distress (%)	26 (27.4)	17 (28.3)	9 (25.7)	0.97
Chest pain (%)	8 (8.4)	5 (8.3)	3 (8.6)	1
Imaging manifestations				
Pulmonary involvement (%)	89 (93.7)	60 (100.0)	29 (82.9)	0.004
Bilateral involvement (%)	89 (93.7)	60 (100.0)	29 (82.9)	0.004
Ground glass opacity (%)	59 (62.1)	44 (73.3)	15 (42.9)	0.006
Patchy consolidation (%)	50 (52.6)	39 (65.0)	11 (31.4)	0.003
Nodules masses (%)	60 (63.2)	39 (65.0)	21 (60.0)	0.79
Halo sign (%)	12 (12.6)	10 (16.7)	2 (5.7)	0.219
Reversed halo sign (%)	12 (12.6)	8 (13.3)	4 (11.4)	1
Air crescent sign (%)	16 (16.8)	13 (21.7)	3 (8.6)	0.174
Cavity (%)	10 (10.5)	6 (10.0)	4 (11.4)	1
Pleural effusion (%)	44 (46.3)	34 (56.7)	10 (28.6)	0.015
Pleural thickening or adhesion (%)	22 (23.2)	14 (23.3)	8 (22.9)	1
Respiratory support (%)				0.007
Invasive mechanical ventilation	39 (41.1)	30 (50.0)	9 (25.7)	
Nasal cannula or mask oxygen therapy	31 (32.6)	19 (31.7)	12 (34.3)	
Non-invasive ventilation	8 (8.4)	6 (10.0)	2 (5.7)	
Non-oxygenated	17 (17.9)	5 (8.3)	12 (34.3)	
PaO_2_/FiO_2_ (mmHg), (median [IQR])	200.00 (128.69, 450.00)	167.10 (85.02, 262.88)	450.00 (234.80, 499.00)	<0.001
SOFA scores (median [IQR])	9.00 (4.50, 14.00)	11.00 (8.00, 14.25)	4.00 (1.00, 9.50)	<0.001
Neutropenia (%)	13 (13.7)	7 (11.7)	6 (17.1)	0.66
Hospitalization (days), (median [IQR])	8.00 (3.00, 35.50)	13.00 (4.00, 40.75)	5.00 (2.00, 32.50)	0.048
ICU admission (%)	51 (53.7)	40 (66.7)	11 (31.4)	0.002
ICU length of stay (days), (median [IQR])	3.00 (0.00, 16.00)	5.00 (0.00, 21.50)	0.00 (0.00, 3.00)	0.001
28th day death (%)	49 (51.6)	39 (65.0)	10 (28.6)	0.001
Laboratory findings				
Hemoglobin (g/L), (median [IQR])	88.00 (72.50, 109.50)	86.00 (71.75, 99.50)	101.00 (73.00, 117.50)	0.102
Platelet count (×10^9^/L), (median [IQR])	87.00 (35.00, 173.50)	73.00 (33.00, 150.75)	119.00 (54.00, 227.50)	0.172
White blood cell count (×10^9^/L), (median [IQR])	8.54 (5.06, 13.27)	9.18 (5.53, 13.48)	7.49 (1.92, 11.61)	0.143
Neutrophil percentage, (median [IQR])	86.00 (73.10, 90.95)	87.55 (77.95, 92.00)	79.90 (70.70, 87.65)	0.017
Neutrophils (×10^9^/L), (median [IQR])	7.15 (3.57, 10.82)	7.28 (4.38, 11.91)	5.80 (1.58, 10.15)	0.107
Lymphocytes (×10^9^/L), (median [IQR])	0.48 (0.23, 0.83)	0.48 (0.23, 0.72)	0.45 (0.23, 1.13)	0.532
LDH (IU/L), (median [IQR])	322.00 (230.50, 564.00)	417.00 (300.00, 631.00)	234.00 (175.00, 280.50)	<0.001
ALB (g/L), (mean [SD])	32.90 (28.80, 36.70)	30.90 (27.40, 34.50)	35.80 (32.20, 38.60)	<0.001
GLB (g/L), (median [IQR])	23.91 (6.42)	22.91 (6.43)	25.64 (6.12)	0.045
TBIL (µmol/L), (median [IQR])	13.30 (8.65, 27.85)	14.60 (9.00, 54.20)	12.00 (8.10, 15.30)	0.122
ALT (IU/L), (median [IQR])	31.00 (15.00, 86.50)	49.00 (15.75, 132.25)	20.00 (14.50, 46.50)	0.065
AST (IU/L), (median [IQR])	45.00 (22.00, 88.50)	53.50 (28.25, 93.75)	24.00 (16.50, 65.50)	0.006
Scr (µmol/L), (median [IQR])	85.00 (58.50, 126.50)	96.00 (65.50, 199.00)	66.00 (47.50, 90.50)	0.001
eGFR (mL/min/1.73m^2^), (median [IQR])	79.20 (46.67, 108.53)	67.13 (33.44, 94.69)	101.28 (82.16, 112.05)	<0.001
BNP (ng/L), (median [IQR])	821.00 (187.50, 3,544.50)	1,084.50 (448.00, 5,037.25)	268.00 (100.50, 2,011.50)	0.009
PCT (ng/mL), (median [IQR])	0.84 (0.30, 3.57)	1.05 (0.44, 4.38)	0.54 (0.22, 1.62)	0.035
CRP (mg/L), (median [IQR])	96.40 (31.70, 193.50)	98.35 (52.08, 206.75)	96.40 (23.45, 148.50)	0.166
IL-6 (pg/mL), (median [IQR])	99.62 (35.70, 271.70)	128.50 (48.65, 377.45)	75.80 (19.85, 133.70)	0.021

^*a*^ mNGS, metagenomic next-generation sequencing; HSCT, hematopoietic stem cell transplantation; LDH, lactate dehydrogenase; ALB, albumin; GLB, globulin; TBIL, total bilirubin; ALT, alanine aminotransferase; AST, aspartate aminotransferase; Scr, serum creatinine; eGFR, estimated glomerular filtration rate; BNP, B-type natriuretic peptide; PCT, procalcitonin; CRP, C-reactive protein; IL-6, interleukin 6; SD, standard deviation; IQR, interquartile range.

### Pathogen spectrum differences between the two groups

**Fig 2:**
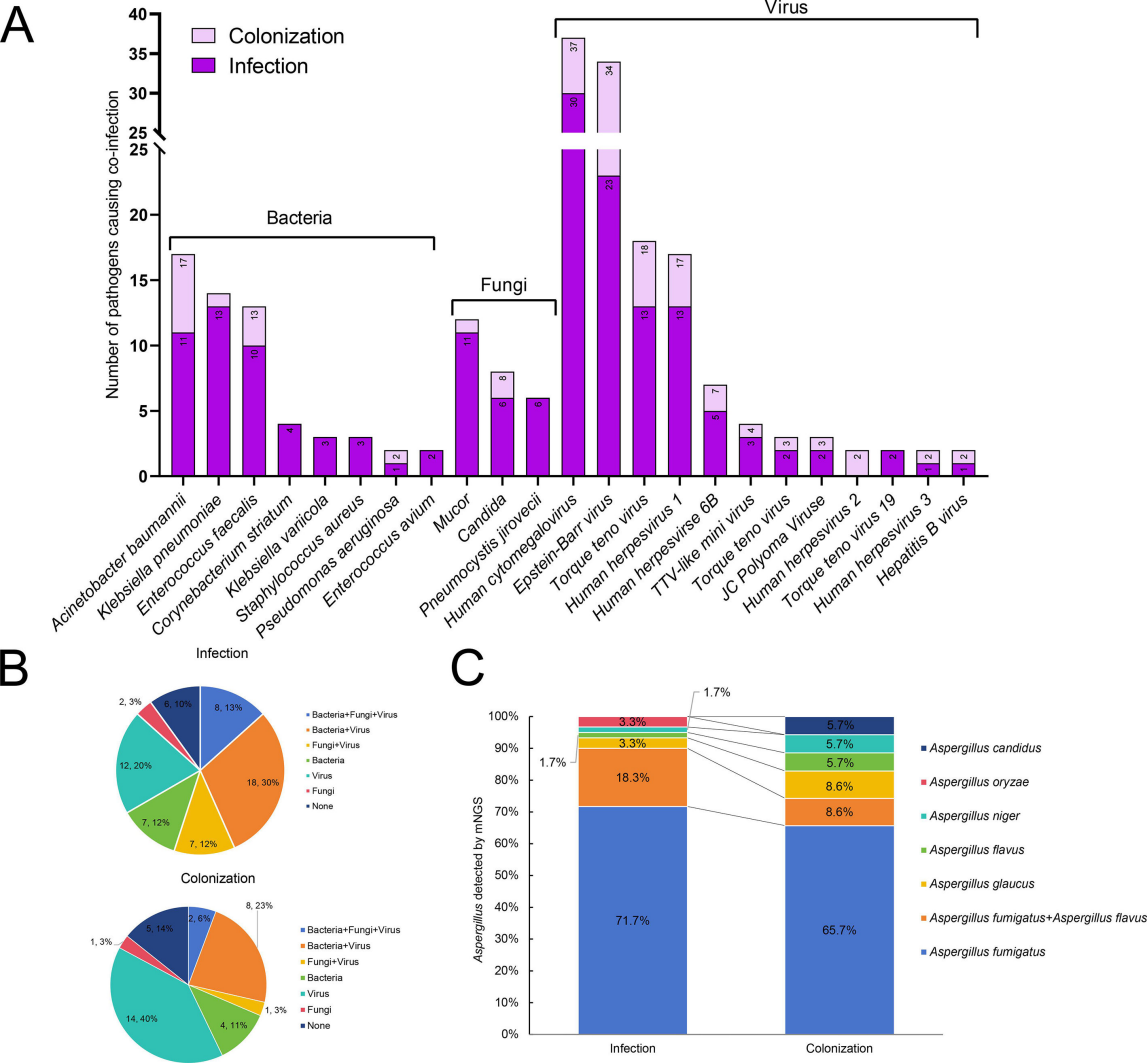
Pathogen spectrum and co-infection patterns. (**A**) Distribution of pathogens co-detected with *Aspergillus* spp. in the colonization (light pink bars) and infection (purple bars) groups. The y-axis represents the number of cases where each pathogen was identified together with *Aspergillus* spp. (**B**) Distribution of microorganism types detected by mNGS within each group. (**C**) Bar chart displaying the distribution of *Aspergillus* species within each group.

Distinct co-infection patterns were observed. Dominant bacterial co-pathogens included *Acinetobacter baumannii* (17/95; infection:9, colonization:6) and *Klebsiella pneumoniae* (14/95; 13:1). Fungal co-infections featured *Mucor* spp. (12/95; 11:1), *Candida* spp. (8/95; 6:2), and *Pneumocystis jirovecii* (6/95; all infection). Viral co-infections predominated with *Human cytomegalovirus* (37/95; 30:7), *Epstein-Barr virus* (34/95; 23:11), and *torque teno virus* (18/95; 13:5; Fig. 2A). Co-infection types diverged: bacteria + virus (infection:18) and bacteria + fungi + virus (infection:8) characterized the infection group, whereas virus alone dominated colonization (14/35; Fig. 2B). A total of six *Aspergillus* species were identified. *Aspergillus fumigatus* constituted 69.5% (66/95) of isolates, with higher infection-group prevalence (71.7% vs. 65.7%). *A. fumigatus + A. flavus* co-detection was more frequent in infection (18.3% vs. 8.6%; Fig. 2C).

### Diagnostic performance of mNGS and serum biomarkers

**Fig 3:**
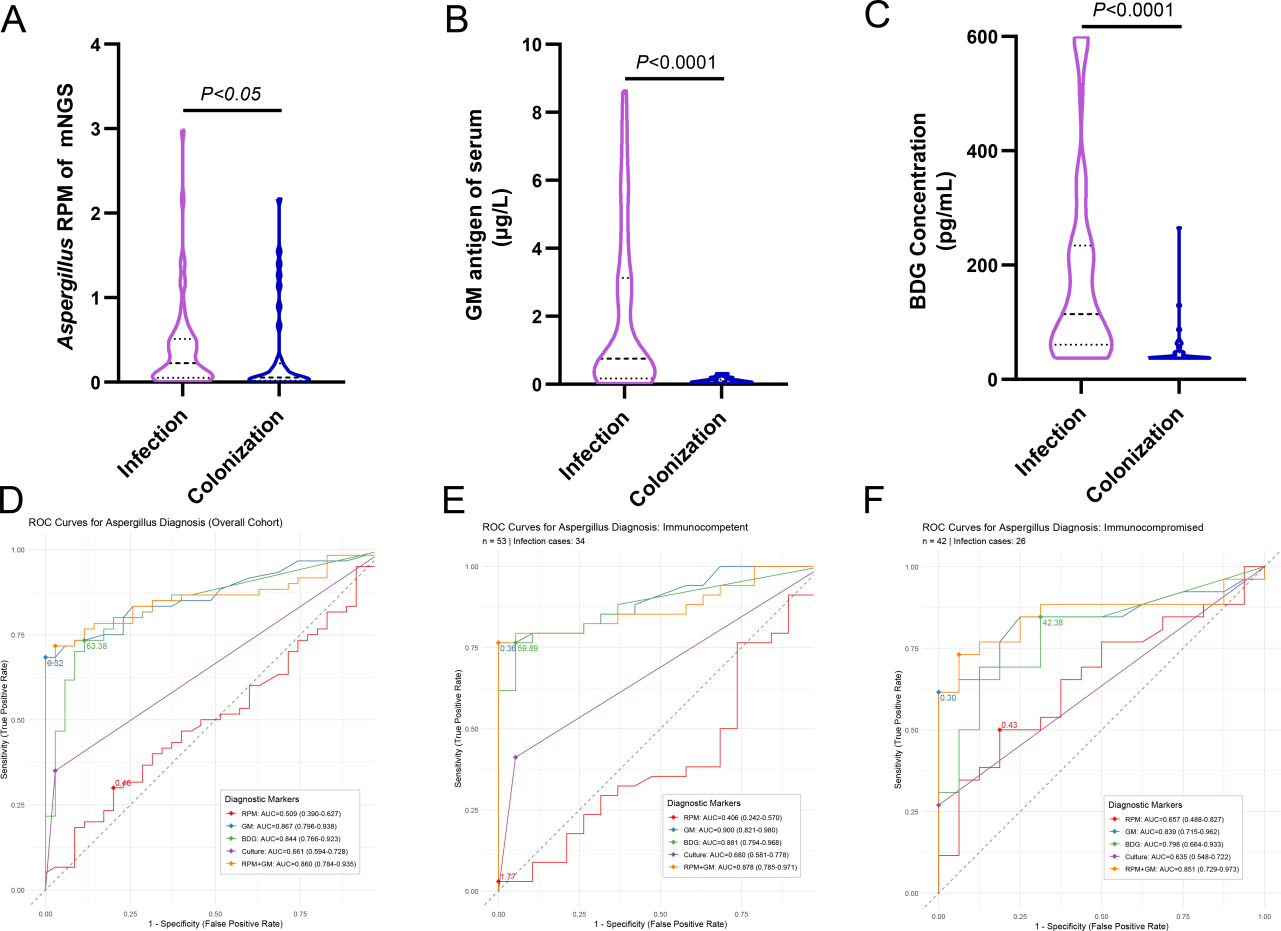
Diagnostic performance of serum biomarkers and mNGS. (**A–C**) Comparison of (**A**) RPM, (**B**) GM, and (**C**) BDG between groups. (**D–F**) ROC curves for diagnosing infection in the (**D**) overall, (**E**) immunocompetent, and (**F**) immunocompromised cohorts. The area under the curve (AUC) values with 95% confidence intervals are provided in the inset.

Levels of *Aspergillus*-RPM, serum GM test, and BDG were significantly higher in the infection group compared to the colonization group (all *P* < 0.05; Fig. 3A through C). ROC curve analysis revealed substantial discriminatory power of these biomarkers for diagnosing *Aspergillus* infection. In the overall cohort (Fig. 3D), both GM test (AUC = 0.867, 95% CI: 0.796–0.938; cutoff: 0.32) and BDG (AUC = 0.844, 95% CI: 0.766–0.923; cutoff: 63.38) outperformed RPM (AUC = 0.509, 95% CI: 0.390–0.627; cutoff: 0.46). The combination of RPM and GM tests achieved an AUC of 0.860 (95% CI: 0.784–0.935). Among immunocompetent patients (Fig. 3E), all biomarkers showed excellent diagnostic performance: GM test (AUC = 0.900, 95% CI: 0.821–0.980; cutoff: 0.36), BDG (AUC = 0.881, 95% CI: 0.794–0.968; cutoff: 59.89), RPM (AUC = 0.406, 95% CI: 0.242–0.570; cutoff: 1.77), and the combination of RPM and GM test (AUC = 0.878, 95% CI: 0.785–0.971). In immunocompromised patients (Fig. 3F), GM test (AUC = 0.839, 95% CI: 0.715–0.962; cutoff: 0.3) and BDG (AUC = 0.798, 95% CI: 0.664–0.933; cutoff: 42.38) retained diagnostic value, whereas RPM performed poorly (AUC = 0.657, 95% CI: 0.488–0.827; cutoff: 0.43). Across all subgroups, fungal culture consistently demonstrated lower diagnostic accuracy, with AUC values ranging from 0.635 to 0.680.

### Correlation analysis between blood mNGS-detected RPM of *Aspergillus* and clinical indicators

**Fig 4:**
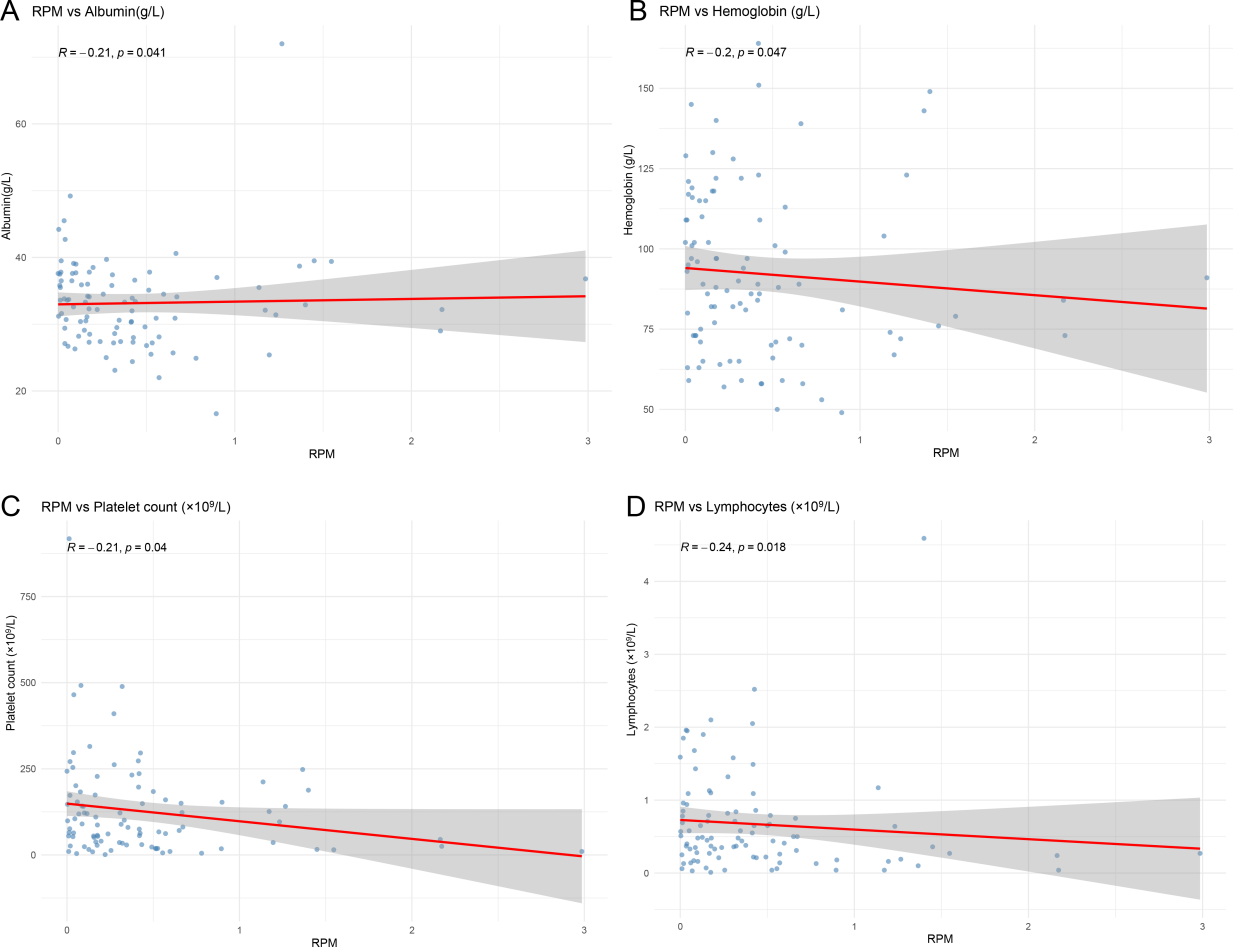
Significant negative correlations between blood mNGS-derived *Aspergillus* load (RPM) and key clinical parameters. Scatter plots illustrating the inverse relationships between RPM values and albumin levels (**A**), hemoglobin concentrations (**B**), platelet counts (**C**), and lymphocyte counts (**D**).

To evaluate the relationship between the fungal load of *Aspergillus* detected by blood mNGS, quantified as RPM, Spearman’s rank correlation analysis was performed on a series of routine clinical parameters (Table 2). Higher *Aspergillus* loads were significantly associated with lower albumin levels (*R* = −0.210, *P* = 0.041), lower hemoglobin levels (*R* = −0.204, *P* = 0.047), lower platelet counts (*R* = −0.211, *P* = 0.040), and lower lymphocyte counts (*R* = −0.243, *P* = 0.018; Fig. 4A and B). In contrast, no statistically significant correlations were observed between RPM values and the oxygenation index (PO_2_/FiO_2_), SOFA score, hepatic and renal function markers (LDH, TBIL, ALT, AST, Scr, and eGFR), B-type natriuretic peptide, white blood cell count, neutrophil count, or inflammatory markers (PCT, CRP, and IL-6; all *P* > 0.05).

**TABLE 2:** Correlations between blood mNGS *Aspergillus* RPM values and clinical parameters^*a*^

Variable	Correlation	*P* value
PO2/FiO2 (mmHg)	−0.058	0.5791
SOFA scores	0.16	0.1226
LDH (IU/L)	0.06	0.5642
ALB (g/L)	−0.21	0.041
GLB (g/L)	−0.108	0.2959
TBIL (µmol/L)	0.135	0.1934
ALT (IU/L)	0.121	0.2425
AST (IU/L)	0.078	0.4497
Scr (µmol/L)	0.129	0.2127
eGFR (mL/min/1.73 m^2^)	−0.122	0.24
B-type natriuretic peptide (ng/L)	−0.027	0.7925
Hemoglobin (g/L)	−0.204	0.0473
Platelet count (×10^9^/L)	−0.211	0.0399
White blood cell count (×10^9^/L)	−0.174	0.0922
Neutrophil percentage	−0.136	0.1895
Neutrophils (×10^9^/L)	−0.199	0.0537
Lymphocytes (×10^9^/L)	−0.243	0.0175
PCT (ng/mL)	0.051	0.6264
CRP (mg/L)	0.026	0.7989
IL-6 (pg/mL)	0.066	0.5227

^*a*^ LDH, lactate dehydrogenase; ALB, albumin; GLB, globulin; TBIL, total bilirubin; ALT, alanine aminotransferase; AST, aspartate aminotransferase; Scr, serum creatinine; eGFR, estimated glomerular filtration rate; PCT, procalcitonin; CRP, C-reactive protein; IL-6, interleukin 6.

### Lasso-Cox regression analysis of 28-day mortality

**Fig 5:**
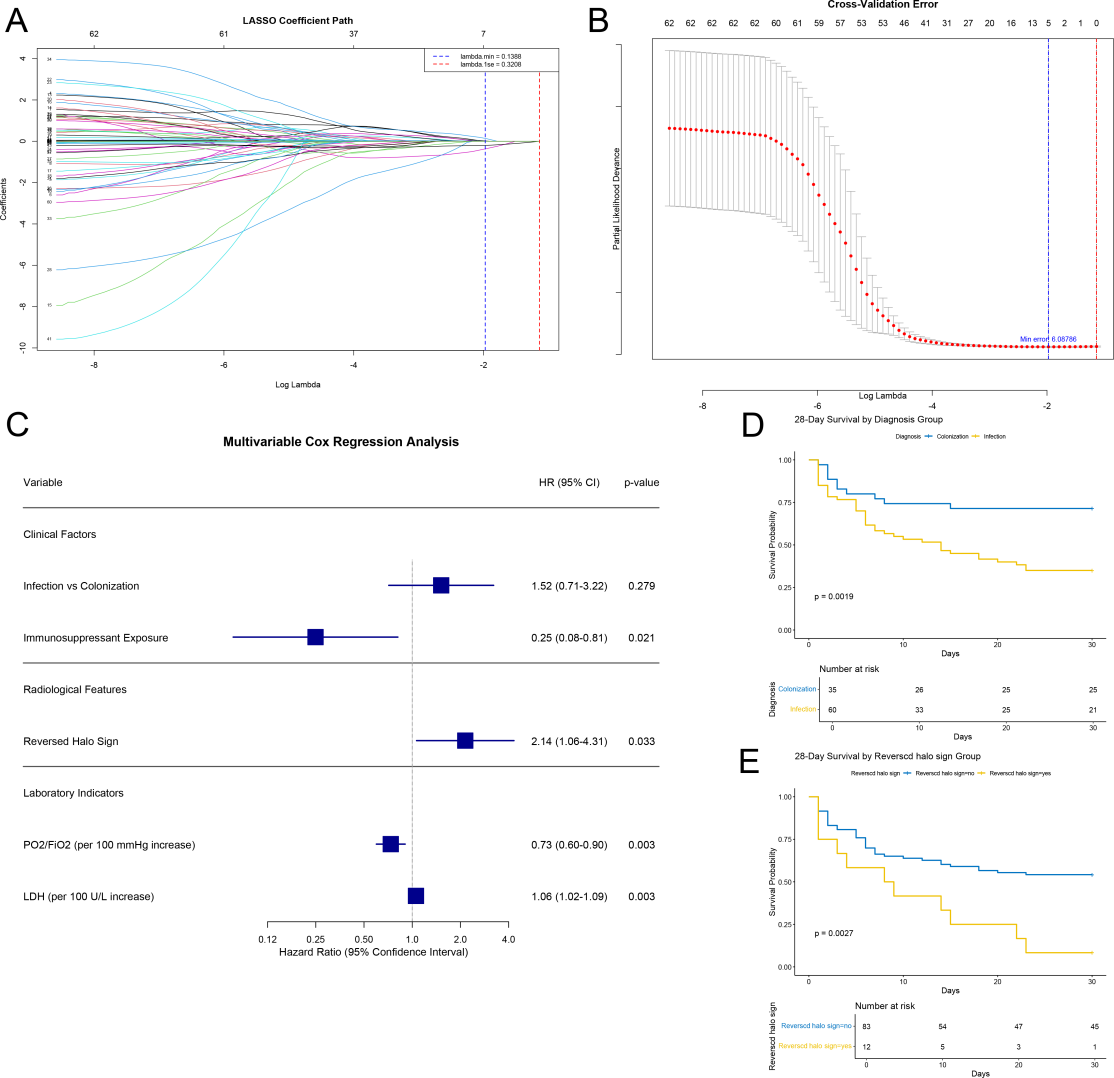
Prognostic analysis of 28-day mortality. (**A**) LASSO coefficient profiles of clinical variables across the log(λ) sequence. (**B**) Variable selection in the LASSO regression using 10-fold cross-validation. The dotted vertical lines indicate the optimal lambda values (lambda.min was selected). (**C**) Forest plot of the final multivariate Cox proportional hazards model, showing hazard ratios (HR) and 95% confidence intervals for the predictors selected by LASSO. (**D**) Kaplan-Meier survival by group (log-rank test, *P* < 0.01). (**E**) Kaplan-Meier survival by reversed halo sign (log-rank test, *P* < 0.01).

Variable selection was performed using LASSO regression with 10-fold cross-validation. Applying the lambda.min criterion, which retains a broader set of predictors, five variables were identified: infection group status, immunosuppressant exposure, reversed halo sign, PaO_2_/FiO_2_ ratio, and LDH level (Fig. 5A and B). These variables were subsequently included in a multivariate Cox proportional hazards model (Fig. 5C). Cox regression analysis identified four independent factors significantly associated with 28-day mortality. Immunosuppressant exposure was protective (HR = 0.249, 95% CI: 0.076–0.813, *P* = 0.021). In contrast, the presence of a reversed halo sign (HR = 2.143, 95% CI: 1.065–4.314, *P* = 0.033), a decrease in the PaO_2_/FiO_2_ ratio (per 100 mmHg reduction; HR = 1.361, 95% CI: 1.107–1.674, *P* = 0.003), and elevated LDH levels (per 100 U/L increase; HR = 1.055, 95% CI: 1.018–1.094, *P* = 0.003) were significant risk factors. Although infection group status was selected by LASSO, it did not retain independent significance in the multivariate Cox model (HR = 1.516, *P* = 0.279). Consistent with the clinical relevance of infection status, Kaplan-Meier analysis demonstrated significantly lower 28-day survival probabilities among patients in the infection group compared to the colonization group (log-rank *P* < 0.01; Fig. 5D). Similarly, the presence of a reversed halo sign was also associated with markedly reduced survival (log-rank *P* < 0.01; Fig. 5E).

## DISCUSSION

This study provides novel insights into the diagnostic and prognostic stratification of patients with *Aspergillus* DNA detected in blood via mNGS. We demonstrate that although blood mNGS effectively identifies *Aspergillus*-derived genetic material, it lacks sufficient specificity to differentiate infection from colonization without integration with clinical, radiological, and complementary biomarker data. To our knowledge, this is the first sizable cohort study designed to distinguish IPA from colonization using blood mNGS. Furthermore, we performed a multivariate survival analysis to identify prognostic factors in this patient population.

The progression from inhalation of *Aspergillus* conidia to active disease depends on a combination of factors, including inoculum size, host immune status, and prior antifungal exposure. Patients with chronic obstructive pulmonary disease, particularly those with severe impairment, are well established as a high-risk group for invasive aspergillosis (19). Other recognized risk factors include advanced age, low body weight, squamous cell carcinoma, tuberculosis or nontuberculous mycobacterial co-infection, diabetes mellitus, systemic corticosteroid use, broad-spectrum antibiotic treatment, and severe viral infections such as influenza (20).

Interestingly, although conventional risk factors for IPA, including specific underlying diseases, immunosuppressant use, or neutropenia, did not significantly differ between the colonization and infection groups in our cohort, clear distinctions emerged in clinical severity and radiological presentation. This suggests that in patients with *Aspergillus* detected by blood mNGS, the transition from colonization to infection may represent a continuous clinical spectrum rather than a binary state defined solely by traditional risk factors.

Notably, the infection group exhibited significantly higher rates of hypoxemia (95.0% vs. 65.7%, *P* < 0.001) and more severe pulmonary imaging features, including bilateral involvement, ground-glass opacities, consolidations, and pleural effusions (all *P* < 0.05). These patients also endured longer hospital and ICU stays and higher ICU admission rates, reflecting greater disease severity. These findings align with the concept that invasive infection develops along a continuum of host–fungal interaction. By comprehensively sequencing all microbial nucleic acids in a sample, blood mNGS can detect pathogens without prior suspicion (21). This capability allows for the identification of *Aspergillus* at an early stage, often before a clinical infection is apparent, highlighting its potential for early intervention in high-risk patients prior to overt deterioration. The ability of blood mNGS to detect *Aspergillus* at an early stage, even before full-blown clinical infection, underscores its potential role in identifying patients along this spectrum prior to overt deterioration.

Blood is considered a sterile site and is not normally colonized by fungal pathogens. Therefore, detection of a fungus in blood typically indicates an invasive fungal infection (22). It is also important to note that clinical specimens can occasionally become contaminated during processing if the laboratory environment is not adequately decontaminated, which may lead to false-positive results (23). Consequently, while a positive blood mNGS result for *Aspergillus* raises strong suspicion for invasive aspergillosis, it must be interpreted with caution. Strict adherence to standardized laboratory workflows, including the use of negative controls and environmental monitoring, is essential to minimize contamination risk and ensure the reliability of mNGS data (24). Positive findings should always be corroborated with clinical, radiological, and other biomarker evidence to avoid misdiagnosis and overtreatment.

*A. fumigatus* was the predominant species across both groups, consistent with its well-established role as the most common pathogenic species in humans (25). The higher frequency of *A. fumigatus* and *A. fumigatus + A. flavus* co-detection in the infection group may suggest a synergistic or compounding pathogenic effect, although the small sample size of non-fumigatus species limits definitive conclusions regarding species-specific virulence in this cohort.

The observed co-infection profiles revealed meaningful pathobiological patterns. The infection group was characterized by a higher prevalence of polymicrobial infections, particularly bacterial-viral and bacterial-fungal-viral complexes, involving pathogens such as *A. baumannii*, *K. pneumoniae*, and *Mucor*, which are clinically significant and potentially pathogenic. This aligns with the state of immunoparalysis seen in invasive fungal disease, where impaired neutrophil and macrophage function facilitates secondary infections (26). *Pneumocystis jirovecii* was detected exclusively in the infection group, underscoring its association with severe T-cell dysfunction, a well-established risk factor for invasive aspergillosis (27).

The higher median RPM observed in the infection group may reflect a greater fungal burden and potential invasiveness. It should be noted, however, that the thick cell wall of fungi can reduce nucleic acid extraction efficiency due to suboptimal breakage, and variations in laboratory protocols may further influence mNGS performance across different fungal species and sample types (28). Our evaluation of the diagnostic performance of mNGS in distinguishing IPA from colonization yielded a critical and nuanced finding: although blood mNGS successfully detected *Aspergillus*, the RPM value alone demonstrated limited ability to differentiate infection from colonization (AUC: 0.5–0.65). This pivotal observation underscores a fundamental principle that the detection of microbial nucleic acid in blood is not synonymous with invasive disease and highlights the paramount importance of clinical context. In contrast, serum biomarkers GM and BDG, which detect fungal cell wall components released during active invasion, exhibited superior diagnostic accuracy (AUCs 0.80–0.90). This suggests that the presence of the fungus (detected by mNGS) and biological evidence of its invasion (detected by GM/BDG) provide complementary information (29, 30).

Notably, our findings align with and extend prior research on mNGS in aspergillosis. While BALF-based mNGS has shown higher discriminatory power for IPA in some cohorts—for example, Xu et al. reported an AUC of 0.894 using an optimal threshold of 23 RPTM in BALF (3), and another study in hematological patients demonstrated superior performance of BALF mNGS compared to serum GM and PCR (31)—blood mNGS offers a non-invasive alternative for critically ill patients unable to undergo bronchoscopy. The lower AUC of RPM alone in our immunocompetent subgroup (0.406) reflects its limited standalone specificity; however, when integrated with GM or BDG, diagnostic performance improved markedly (AUC up to 0.900), justifying its characterization as part of an “excellent” multimodal panel rather than as a standalone test. This continuum of diagnostic utility across sample types underscores the concept of a “continuous clinical spectrum” in aspergillosis: BALF mNGS may better capture localized pulmonary invasion, whereas blood mNGS, especially when combined with serological biomarkers, reflects systemic dissemination and host response. Such integration is essential for accurate stratification along the colonization–infection continuum.

The correlation analysis offers a compelling pathophysiological explanation for the above findings. The lack of correlation between RPM and traditional inflammatory markers (PCT, CRP, and IL-6) or the SOFA score is highly instructive. It suggests that a high *Aspergillus* load in the bloodstream does not necessarily trigger a proportionate systemic inflammatory response. Instead, the significant negative correlations with albumin, hemoglobin, platelets, and lymphocytes paint a picture of consumption, bone marrow suppression, and a state of immune exhaustion. Therefore, a high RPM value may be less an indicator of acute inflammation and more a biomarker of the chronic hematological and nutritional burden of the fungal disease. This explains why it lacks specificity for acute invasion but is associated with a poorer overall clinical state.

The prognostic analysis identified key mortality determinants through LASSO-Cox regression. The reversed halo sign emerged as a significant radiological predictor (HR = 2.143), consistent with its association with angioinvasive disease and treatment resistance. Hypoxemia (PaO_2_/FiO_2_ reduction per 100 mmHg; HR = 1.361) and elevated LDH (per 100 U/L increase; HR = 1.055) were independent risk factors, reflecting disease severity and cellular damage from fungal invasion (32). Paradoxically, immunosuppressant exposure conferred protection (HR = 0.249), likely reflecting appropriate management of underlying conditions rather than a direct biological effect. While infection group status showed strong association with mortality in univariate analysis (65.0% vs. 28.6%), it did not retain independent prognostic significance in the multivariate model, emphasizing that the identified risk triad—radiological signs, respiratory failure, and tissue damage—transcends diagnostic categorization (33).

Several limitations warrant consideration. The retrospective design introduces potential selection bias, and the single-center cohort may limit generalizability. The modest sample size constrained subgroup analyses, particularly for rare *Aspergillus* species. Future multicenter studies should validate these findings, explore host transcriptional signatures to complement pathogen detection, and assess how integrating mNGS with biomarker kinetics affects early diagnosis.

### Conclusion

This study confirms that blood mNGS, while sensitive for *Aspergillus* detection, cannot alone distinguish infection from colonization. The clinical value of mNGS-derived fungal load lies in its correlation with host immune and nutritional status rather than acute invasiveness, offering prognostic insight into chronic disease burden. Diagnostic accuracy significantly improves when mNGS is combined with serological biomarkers (GM and BDG) and clinical-radiological assessment. Key mortality predictors included reversed halo sign, hypoxemia, and elevated LDH. These findings support a multimodal diagnostic strategy to guide aspergillosis management and early intervention.

## Data Availability

The data set used and analyzed during the current study is available from the corresponding author on reasonable request.

## SUPPLEMENTAL MATERIAL

Table S1 spectrum.03384-25-s0001.doc Diagnostic details for each patient.

ASM does not own the copyrights to Supplemental Material that may be linked to, or accessed through, an article. The authors have granted ASM a non-exclusive, world-wide license to publish the Supplemental Material files. Please contact the corresponding author directly for reuse.
