# Supplementary material for: The diagnostic and prognostic utility of blood metagenomic next-generation sequencing for invasive pulmonary aspergillosis
Source: Microbiol Spectr. 2026 Apr 17;14(6):e03384-25. doi: 10.1128/spectrum.03384-25 (PMC13228001; doi:10.1128/spectrum.03384-25)
Supplement: Table S1 — Diagnostic details for each patient. [file spectrum.03384-25-s0001.doc]

Table S1 Diagnostic details for each patient

| Patient ID | Proven evdience | Probable evdience | | | EORTC/MSGERC diagnosis | Final diagnosis |
| --- | --- | --- | --- | --- | --- | --- |
|  |  | Host factors | Clinical features | Mycological evidence |  |  |
| 1 | 0 | 0 | 0 | 0 | Unlikely | Colonization |
| 2 | 0 | 0 | 0 | 0 | Unlikely | Colonization |
| 3 | 1 | 1 | 1 | 1 | Proven | Infection |
| 4 | 0 | 1 | 0 | 0 | Unlikely | Colonization |
| 5 | 0 | 1 | 1 | 1 | Probable | Infection |
| 6 | 0 | 1 | 1 | 0 | Possible | Infection |
| 7 | 0 | 1 | 0 | 0 | Unlikely | Colonization |
| 8 | 0 | 1 | 0 | 0 | Unlikely | Colonization |
| 9 | 0 | 1 | 0 | 0 | Unlikely | Colonization |
| 10 | 0 | 0 | 1 | 0 | Unlikely | Colonization |
| 11 | 0 | 1 | 1 | 0 | Possible | Infection |
| 12 | 0 | 0 | 1 | 1 | Possible | Infection |
| 13 | 0 | 0 | 1 | 0 | Unlikely | Colonization |
| 14 | 0 | 1 | 1 | 0 | Possible | Infection |
| 15 | 0 | 1 | 1 | 0 | Possible | Infection |
| 16 | 0 | 1 | 1 | 0 | Possible | Infection |
| 17 | 1 | 1 | 1 | 1 | Proven | Infection |
| 18 | 0 | 1 | 1 | 1 | Probable | Infection |
| 19 | 0 | 0 | 0 | 0 | Unlikely | Colonization |
| 20 | 0 | **1** | **1** | 0 | Possible | Infection |
| 21 | 0 | 1 | 1 | 1 | Probable | Infection |
| 22 | 0 | 1 | 1 | 0 | Possible | Infection |
| 23 | 0 | 0 | 0 | 0 | Unlikely | Colonization |
| 24 | 0 | 0 | 1 | 0 | Unlikely | Colonization |
| 25 | 0 | 0 | 1 | 0 | Unlikely | Colonization |
| 26 | 0 | 0 | 1 | 0 | Unlikely | Colonization |
| 27 | 0 | **0** | **1** | 0 | Unlikely | Colonization |
| 28 | 0 | 1 | 1 | 1 | Probable | Infection |
| 29 | 0 | 1 | 1 | 0 | Possible | Infection |
| 30 | 0 | 1 | 1 | 1 | Probable | Infection |
| 31 | 0 | 1 | 1 | 1 | Probable | Infection |
| 32 | 1 | 1 | 1 | 0 | Proven | Infection |
| 33 | 0 | **1** | **1** | 0 | Possible | Infection |
| 34 | 0 | 1 | 1 | 1 | Probable | Infection |
| 35 | 0 | 1 | 1 | 1 | Probable | Infection |
| 36 | 0 | **1** | **1** | 0 | Possible | Infection |
| 37 | 0 | 0 | 0 | 0 | Unlikely | Colonization |
| 38 | 0 | 1 | 1 | 1 | Probable | Infection |
| 39 | 0 | 1 | 1 | 1 | Probable | Infection |
| 40 | 0 | 0 | 1 | 1 | Unlikely | Colonization |
| 41 | 0 | 0 | 1 | 0 | Unlikely | Colonization |
| 42 | 0 | 1 | 1 | 0 | Possible | Infection |
| 43 | 0 | 0 | 1 | 0 | Unlikely | Colonization |
| 44 | 0 | 0 | 1 | 1 | Unlikely | Colonization |
| 45 | 1 | 1 | 1 | 0 | Proven | Infection |
| 46 | 0 | 1 | 1 | 1 | Probable | Infection |
| 47 | 0 | 1 | 1 | 1 | Probable | Infection |
| 48 | 0 | 1 | 1 | 0 | Possible | Infection |
| 49 | 0 | 1 | 1 | 1 | Probable | Infection |
| 50 | 0 | 0 | 1 | 0 | Unlikely | Colonization |
| 51 | 0 | **1** | **1** | 0 | Possible | Infection |
| 52 | 0 | 1 | 1 | 1 | Probable | Infection |
| 53 | 0 | 0 | 0 | 0 | Unlikely | Colonization |
| 54 | 0 | 1 | 1 | 1 | Probable | Infection |
| 55 | 0 | 0 | 1 | 0 | Unlikely | Colonization |
| 56 | 1 | 1 | 1 | 0 | Proven | Infection |
| 57 | 0 | 0 | 1 | 0 | Unlikely | Colonization |
| 58 | 0 | 1 | 1 | 0 | Possible | Infection |
| 59 | 0 | 0 | **1** | 0 | Unlikely | Colonization |
| 60 | 0 | 1 | 1 | 1 | Probable | Infection |
| 61 | 0 | 0 | **1** | 1 | Possible | Infection |
| 62 | 0 | 1 | 1 | 0 | Possible | Infection |
| 63 | 0 | 1 | 1 | 0 | Possible | Infection |
| 64 | 0 | 1 | 1 | 1 | Probable | Infection |
| 65 | 0 | 1 | 1 | 0 | Possible | Infection |
| 66 | 0 | 1 | 1 | 1 | Probable | Infection |
| 67 | 0 | 1 | 0 | 1 | Unlikely | Colonization |
| 68 | 0 | 0 | 0 | 1 | Unlikely | Colonization |
| 69 | 0 | 1 | 1 | 0 | Unlikely | Colonization |
| 70 | 0 | 0 | **1** | 0 | Unlikely | Colonization |
| 71 | 0 | 0 | 1 | 0 | Unlikely | Colonization |
| 72 | 0 | 1 | 1 | 0 | Possible | Infection |
| 73 | 0 | 0 | 1 | 0 | Unlikely | Colonization |
| 74 | 0 | 1 | 1 | 0 | Possible | Infection |
| 75 | 0 | 1 | 1 | 1 | Probable | Infection |
| 76 | 0 | 0 | 0 | 0 | Unlikely | Colonization |
| 77 | 0 | 1 | 1 | 1 | Probable | Infection |
| 78 | 1 | 1 | 1 | 1 | Proven | Infection |
| 79 | 0 | 0 | 0 | 0 | Unlikely | Colonization |
| 80 | 0 | 1 | 1 | 0 | Possible | Infection |
| 81 | 0 | 1 | 1 | 0 | Possible | Infection |
| 82 | 0 | 1 | 1 | 1 | Probable | Infection |
| 83 | 0 | 1 | 1 | 0 | Possible | Infection |
| 84 | 0 | 0 | 1 | 0 | Unlikely | Colonization |
| 85 | 0 | 1 | 1 | 1 | Probable | Infection |
| 86 | 1 | 1 | 1 | 0 | Proven | Infection |
| 87 | 0 | 1 | 1 | 0 | Possible | Infection |
| 88 | 0 | 1 | 1 | 0 | Possible | Infection |
| 89 | 0 | 1 | 1 | 1 | Probable | Infection |
| 90 | 0 | 1 | 1 | 0 | Possible | Infection |
| 91 | 0 | 1 | 1 | 1 | Probable | Infection |
| 92 | 0 | 0 | 1 | 0 | Unlikely | Colonization |
| 93 | 1 | 1 | 1 | 1 | Proven | Infection |
| 94 | 0 | 1 | 1 | 1 | Probable | Infection |
| 95 | 0 | 0 | 1 | 0 | Unlikely | Colonization |
